# Late enhancement and wash-out maps for differentiation of glioblastoma and metastases

**DOI:** 10.1186/s12880-025-01889-6

**Published:** 2025-08-27

**Authors:** Leon Schmidt, Eya Khadhraoui, Stefan Klebingat, I. Erol Sandalcioglu, Roland Schwab, Belal Neyazi, Daniel Behme, Klaus-Peter Stein, Sebastian Johannes Müller

**Affiliations:** 1 Clinic for Neuroradiology, Otto-Von-Guericke-University Magdeburg, Leipziger Str. 44, D-39120 Magdeburg, Germany; 2 Department of Neurosurgery, Otto-Von-Guericke-University Magdeburg, Leipziger Str. 44, D-39120 Magdeburg, Germany; 3 Stimulate Research Campus Magdeburg, Otto-Hahn-Str. 2, D-39106 Magdeburg, Germany

**Keywords:** Late enhancement, Contrast clearance, Wash-out, MR perfusion, Brain metastases, Glioblastoma

## Abstract

**Background:**

Advanced MR imaging methods, such as perfusion, spectroscopy and diffusion-weighted imaging (DWI), help to distinguish between different types of brain tumors. We have established a rapid wash-out and late-enhancement map in our clinic. The aim of our study was to evaluate the potential usefulness of this map for the initial diagnosis.

**Methods:**

We included patients with a histologically confirmed initial diagnosis of brain metastasis and glioblastoma who underwent our MR protocol, including MR perfusion and rapid wash-out imaging, between January 1, 2024, and March 31, 2025. The rapid wash-out and late enhancement maps are derived from two contrast-enhanced T1-weighted 3D datasets acquired at 5 and 25 min after contrast injection. We volumetrically measured the late-enhancement and wash-out of brain tumors at initial diagnosis, and analyzed the wash-out ratio as volume (wash-out) ∶ volume (wash-out + late enhancement). Additionally, we evaluated the relative cerebral blood volume (rCBV) and the apparent diffusion coefficient (ADC) ratios.

**Results:**

Forty-three patients were included in the study: 21 with glioblastoma and 22 with brain metastases. For glioblastoma, the mean wash-out ratio was 0.39 ± 0.21, the ADC ratio 1.41 ± 0.33, and the rCBV ratio 3.49 ± 1.25. In comparison, brain metastases showed a mean wash-out ratio of 0.23 ± 0.15, an ADC ratio of 1.38 ± 0.47, and an rCBV ratio of 2.25 ± 1.55. While rCBV ratio and wash-out ratio exhibited significant differences between glioblastomas and brain metastases, no such differences were observed in ADC ratios. An area-under-curve analysis revealed a ratio of 0.29 as the best threshold (for tumors larger than 1 cm³) for the wash-out ratio to distinguish glioblastomas and brain metastases with a sensitivity of 70% and a specificity of 75%.

**Conclusions:**

The wash-out ratio serves as an additional valuable marker for distinguishing between glioblastoma and brain metastases. Since MR perfusion is not always reliable for this differentiation, this novel method provides additional diagnostic confidence during the initial assessment. Combining rCBV and wash-out ratios may provide higher diagnostic accuracy than using either method individually.

**Clinical trial number:**

Not applicable.

## Introduction

Brain metastases and glioblastoma represent the most common malignant brain tumors encountered in clinical neuroimaging [1, 2]. Accurate differentiation between these entities at the time of initial diagnosis is crucial, as it directly influences treatment planning, prognosis, and patient management [3–5]. Conventional MRI techniques, including T1-weighted imaging with contrast enhancement, T2-weighted sequences, and fluid-attenuated inversion recovery (FLAIR), provide essential morphological insights. However, both tumor types can exhibit overlapping radiological features, such as ring-enhancing lesions, peritumoral edema, and central necrosis, making precise differentiation challenging [6]. Advanced MRI modalities, including diffusion-weighted imaging (DWI), perfusion-weighted imaging (PWI) [7], and magnetic resonance spectroscopy (MRS), have emerged as valuable tools to enhance diagnostic accuracy.

**Fig. 1:**
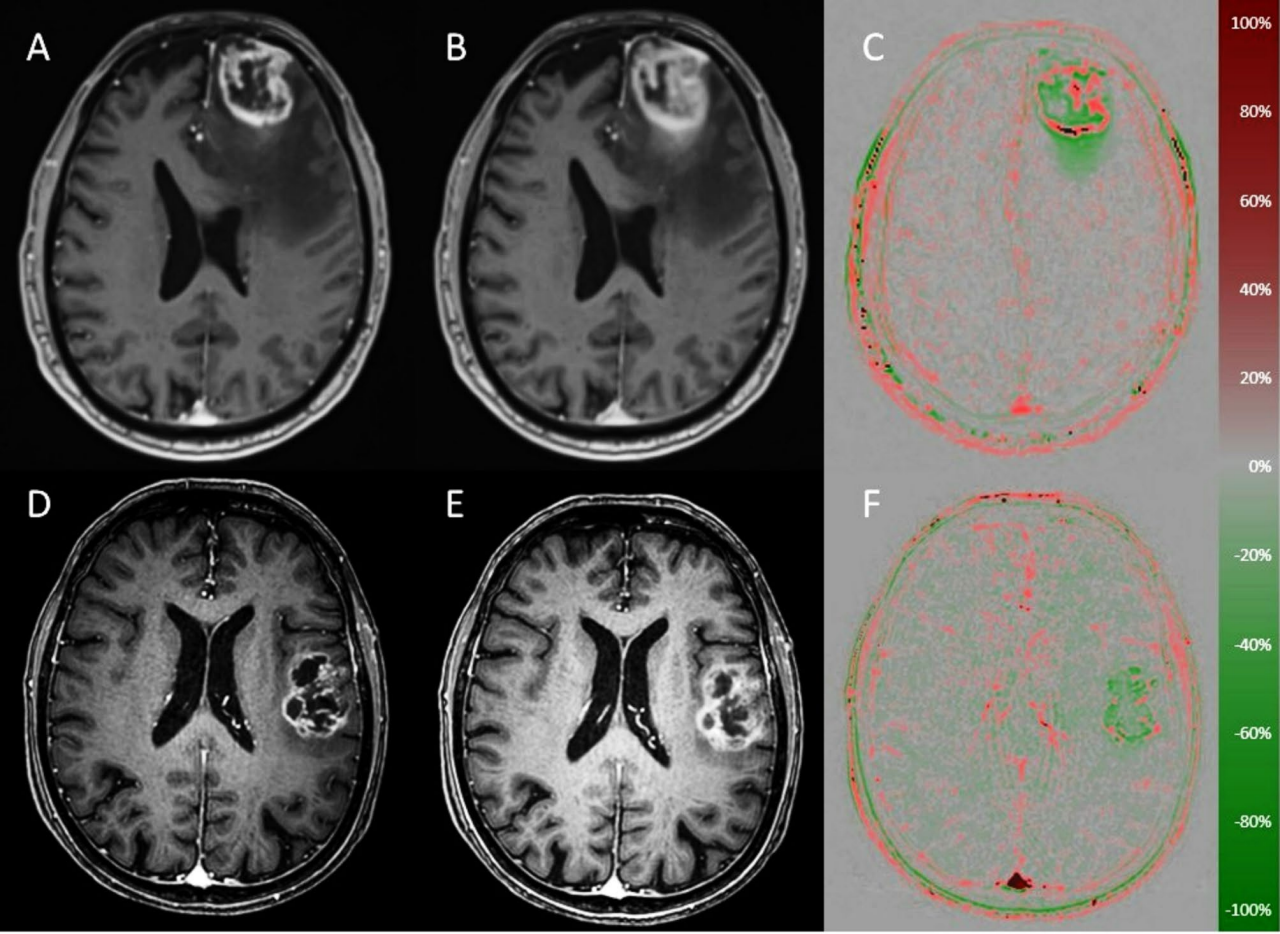
Introduction example. Transversal early and late contrast-enhanced T1-weighted imaging of a patient with glioblastoma (**A-B**) and a patient with a brain metastasis from non-small cell lung cancer (**D-E**). The calculated wash-out maps are illustrated in **C** and **F**. The scale represents wash-out percentages, with negative values indicating late enhancement

Experimental studies suggest that high-grade gliomas cells reach peak contrast enhancement approximately 3–8 min [8, 9] after contrast administration, a property that can be leveraged for improved diagnostic accuracy. Based on this, we optimized our imaging protocol by acquiring 3D T1-weighted sequences at approximately 5 min and again at 25 min post-contrast injection. The initial aim was to better differentiate between progressive disease and radiation necrosis. To this end, we originally developed an MR rapid wash-out map based on two T1-weighted datasets [10]. Figure 1 demonstrates two examples. The principle is straightforward: the two T1-weighted sequences are co-registered, followed by intensity normalization and subtraction. The resulting difference image is then visualized using a red–green color map. Red is used to visualize the wash-out phenomenon, whereas green represents areas of late enhancement.

The aim of the current study was to investigate whether this delayed-enhancement approach could also improve the differentiation between glioblastoma and brain metastases during initial diagnosis.

## Methods

### Study design

This single-center observational study was ethically approved by the institutional review board and adhered to the 2013 Declaration of Helsinki. The institutional review board waived the requirement for informed consent because of the retrospective nature of the study and the analysis of anonymized patient data. All methods were performed in accordance with relevant guidelines and regulations.

### Participant population

We searched our Picture Archiving and Communication System (PACS) for patients with valid rapid wash-out protocols between 01/01/2024 and 31/03/2025. Patients were included if they had undergone a complete MRI tumor protocol at the time of their initial diagnosis of a brain tumor and had either a histologically proven glioblastoma or brain metastasis.

Exclusion criteria were as follows: (1) a neuropathological diagnosis other than brain metastasis or glioblastoma; (2) technical aspects, such as motion artifacts or absence of contrast agent, that rendered the calculation of wash-out maps infeasible; (3) patient age under 18 years; (4) brain tumors that had been previously treated (either irradiated or subjected to targeted chemotherapy). In our study, patients who had received systemic therapy for the primary tumor but no targeted treatment for newly developed cranial metastases during the course of their treatment were not excluded.

### MRI protocol and technical details

MRI protocols were performed on Siemens Sola 1.5T (Siemens Healthineers AG, Munich, Germany), Philips Achieva 3T and Philips Intera 1.5T (Koninklijke Philips NV, Amsterdam, Netherlands). The detailed MR protocols and the description of the wash-out software were published previously [10]. The protocol includes dynamic susceptibility contrast (DSC) MR perfusion without a pre-bolus, as well as echo-planar diffusion-weighted imaging (EPI-DWI). To calculate the wash-out maps, a Python script retrieves both T1-weighted sequences from the PACS. Co-registration is performed using the FLIRT algorithm (FMRIB’s Linear Image Registration Tool) [11–14], followed by intensity normalization and subtraction. The final wash-out maps are then exported back to the PACS system.

### Measurements

All measurements were performed independently by two raters and finally averaged. To assess interrater reliability, the intraclass correlation coefficient ICC(2,k) was calculated using a two-way random-effects model with average measures. We measured wash-out and wash-in (late-enhancement) volumes for every patient via 3D slicer (level trace tool, slice by slice, semi-automatically). We also calculated the

$$\begin{gathered}Wash - outratio \hfill \\= \frac{{Volume\,(wash-out)}}{\begin{gathered}Volume\left({wash-out} \right)\, \hfill \\+ Volume\left({lateenhancement} \right) \hfill \\\end{gathered}} \hfill \\\end{gathered}$$

as described in a TRAMs study [15]. Additionally, we assessed the ADC of solid tumor parts using INFINITT PACS (INFINITT Healthcare, Seoul, South Korea), when possible, as well as the healthy contralateral hemisphere, and calculated a normalized ADC ratio, as described in a study on brain metastases from non-small cell lung cancer and small cell lung cancer [16]. The relative cerebral blood flow (rCBV) of the contrast-enhancing tumor region was measured in three different slices and the rCBV ratio was calculated as described in a previous study [17]. For this purpose, we utilized IntelliSpace Portal software (Philips Healthcare, Eindhoven, The Netherlands).

### Statistical analysis

We used Python3 (Version 3.11, matPlotLib) for statistical programming. Histograms and images were generated using Microsoft Excel (Version 16.0, Microsoft Corporation, Redmond, WA, USA). For group comparisons, an unpaired *t*-test was performed. A significance level of *p* < 0.05 was applied to determine statistical significance. 95% confidence intervals (CI) were calculated.

To rule out a potential spurious correlation between MR perfusion and wash-out, we conducted linear regression analyses using the R statistical software und linear models. Multilevel linear models were performed using the “lm” method of R Version 4.2.2 (https://r-project.org/).

ICC(2,*k*) was calculated using Python3 with the libraries “pandas” and “pingouin” and interpreted according to Koo and Li [18].

To evaluate the diagnostic performance of the test, the Receiver Operating Characteristic (ROC) curve and the corresponding Area Under the Curve (AUC) were used. The optimal threshold was calculated using Youden’ J.

## Results

### Participants

**Fig. 2:**
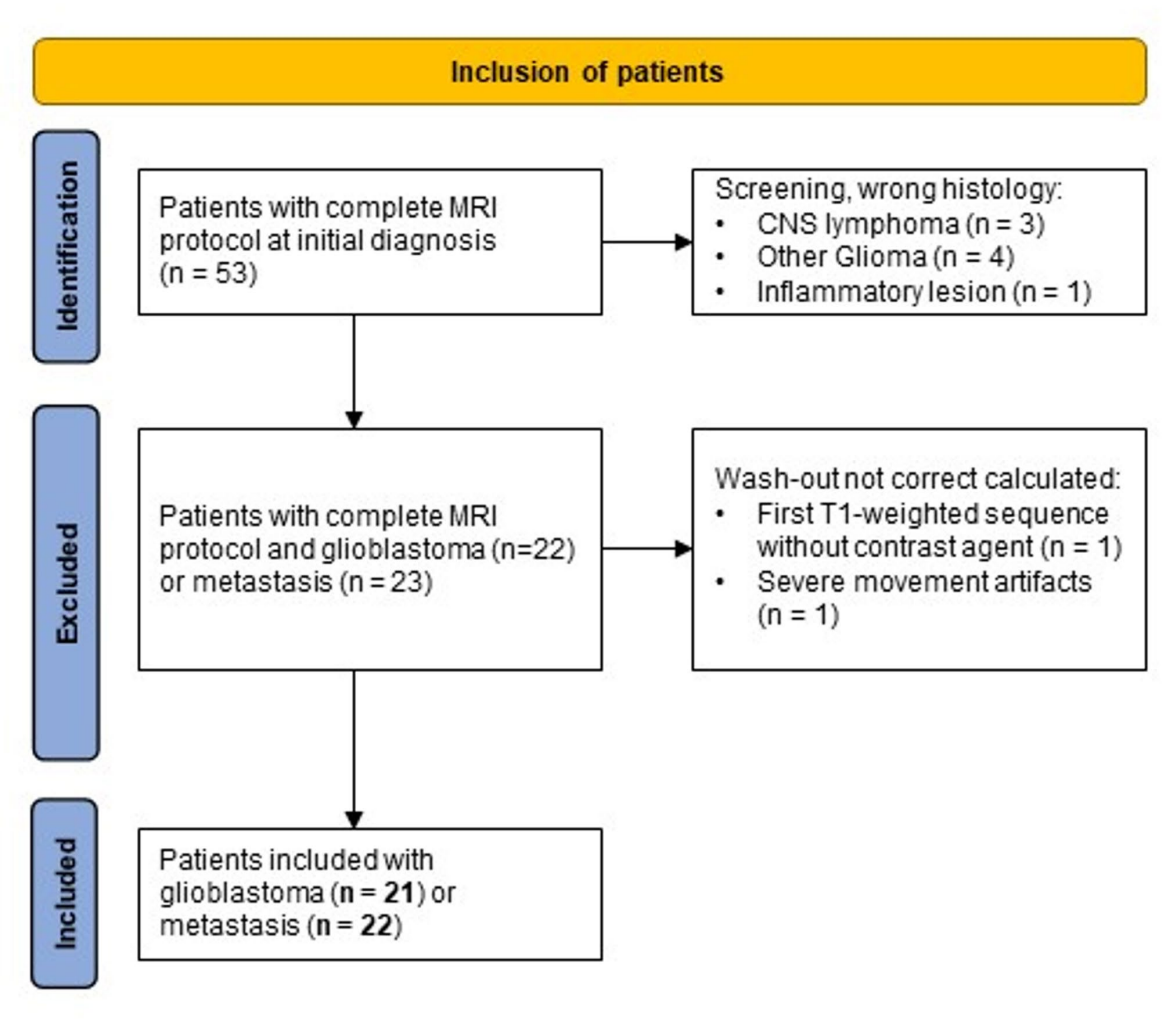
Inclusion flow chart

Our PACS search identified 53 patients with a complete MRI tumor protocol, including rapid wash-out maps. Eight patients were excluded during screening due to incorrect histology, and two additional were excluded because of errors in one T1-weighted sequence (absent contrast enhancement / severe movement artifacts). Ultimately, 43 patients (21 with glioblastoma and 22 with brain metastases) were included. The inclusion flow chart is illustrated in Fig. 2.

The mean age of patients with glioblastoma was 62 ± 11 years (mean ± standard deviation), including 7 female patients, and 65 ± 6 years (9 female patients) for those with brain metastases.

A minimum lesion size of 3 × 3 × 3 mm was established to ensure accurate measurements. In total, we analyzed 29 glioblastoma manifestations and 53 brain metastases.

Mean tumor volumes were 12,992 ± 18,089 mm³ for glioblastoma and 4,488 ± 6,756 mm³ for brain metastasis. Detailed volumes and measurements are listed in Table 1.

### Histopathology

The mutation status of the 21 patients with glioblastoma was as follows: all 21 had isocitrate dehydrogenase (IDH)-wildtype; 11 had a telomerase reverse transcriptase (TERT) promoter C228T mutation, and 10 had an O6-methylguanine-DNA methyltransferase (MGMT) methylation.

The average proliferation index, according to Mindbomb E3 Ubiquitin Protein Ligase 1 (MIB-1) was 18 ± 9%.

Among the 22 patients with brain metastases, primary tumors included 10 non-small cell lung (NSCLC) cancers, six small cell lung (SCLC) cancers, two breast cancers, and one each of renal cell, urothelial, and esophageal carcinoma.

### MR perfusion and rCBV ratio

**Fig. 3:**
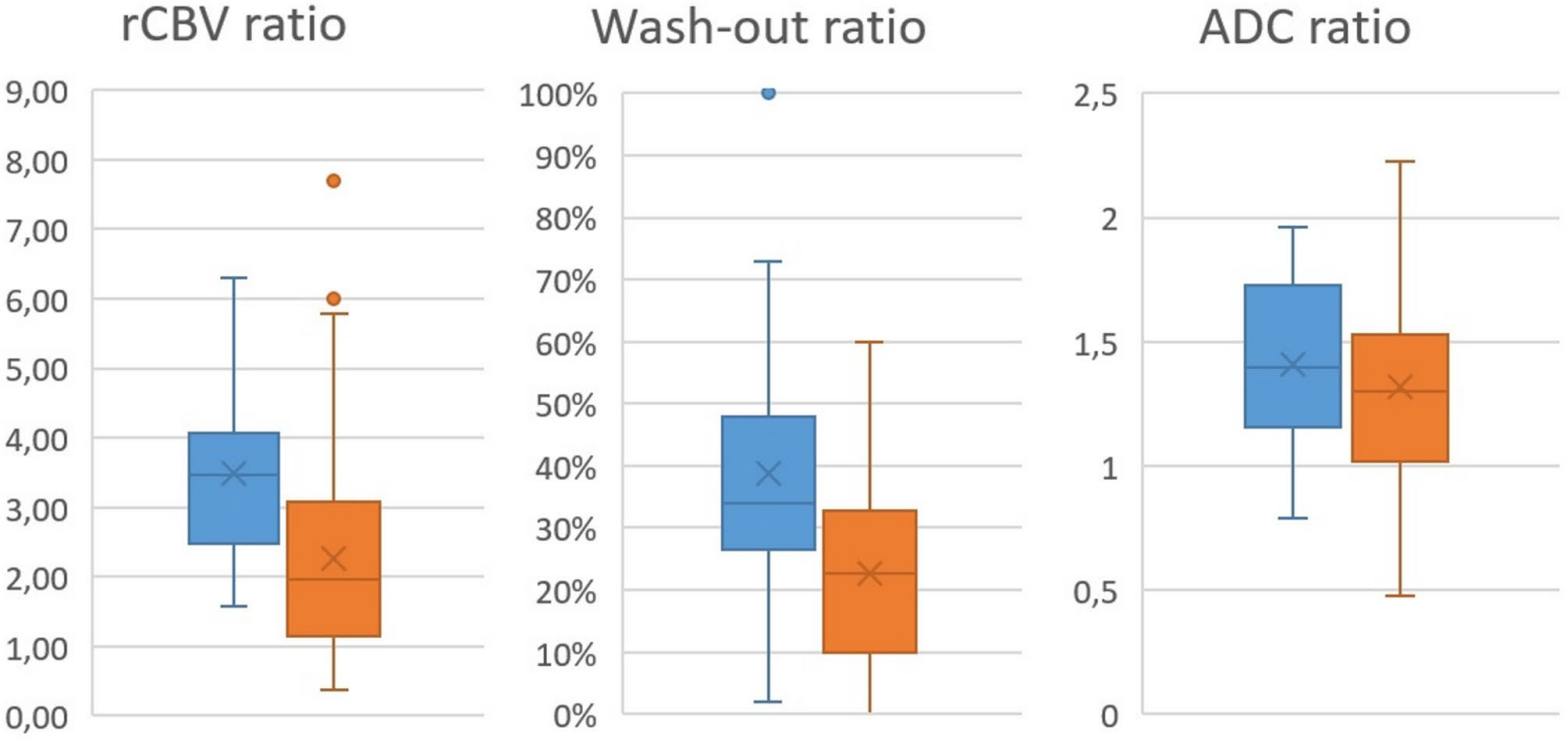
Boxplots of relative cerebral blood volume (rCBV) ratio, wash-out ratio and apparent diffusion coefficient ratio of glioblastoma (blue) and brain metastasis (orange). Box: interquartile range (IQR), which spans from the lower quartile (Q1) to the upper quartile (Q3). Whiskers: Indicate the minimum and maximum data range, excluding outliers. Median line: median. X: arithmetic mean

The mean and standard deviation were 3.49 ± 1.25 for glioblastoma and 2.25 ± 1.55 for brain metastases; both values differed significantly. Figure 3 demonstrates boxplots comparing the analyzed main properties of glioblastoma and brain metastasis.

A ROC AUC analysis revealed an rCBV ratio < 2.23 (Youden’s J 0.53) as the optimal threshold with a sensitivity of 69% and specificity of 84% for brain metastasis compared with glioblastoma (AUC = 0.77, CI 0.65–0.89). The exclusion of small tumors (volume < 1 cm³) decreased the AUC to 0.76 (CI 0.63–0.90).

### Wash-out maps

The mean wash-out ratio was 0.39 ± 0.21 (range 0.02-1.00) for glioblastoma, and 0.23 ± 0.15 (range 0.00-0.60) for brain metastases; both values differed significantly.

The ROC AUC analysis identified a wash-out ratio threshold of < 0.26 as optimal for distinguishing brain metastases from glioblastomas, yielding a sensitivity of 64% and specificity of 79% (Youden’s J 0.43, AUC = 0.72, CI 0.61–0.83). The exclusion of small tumors (volume < 1 cm³) increased the AUC slightly to 0.73 (CI 0.61–0.85, Youden’s J 0.45), with an optimal threshold < 0.29 (sensitivity: 70%; specificity: 75%).

The AUC of the combined product (rCBV ratio $\:\times\:$ wash-out ratio) revealed an optimal sensitivity of 76% and a specificity of 80% with an AUC of 0.79 (CI 0.69–0.89, Youden’s J 0.56; threshold < 0.58). When excluding small tumors, the AUC remained comparable at 0.79 (CI 0.69–0.90).

### ADC ratio

Mean and standard deviation of the ADC ratios were 1.41 ± 0.33 for glioblastoma and 1.38 ± 0.47 for brain metastases; both values were not significantly different.

### Linear models

The linear model “lm (formula = tumor-type ~ wash-out-ratio + rCBV-ratio + ADC-ratio + tumor-volume, data = data)” revealed significant “true” correlations of the tumor type (glioblastoma or metastases) with both rCBV ratio (*p* = 0.026) and the wash-out ratio (*p* < 0.001). No significant association was found for the ADC ratio (*p* = 0.71), whereas tumor volume was also significantly associated with tumor type (*p* = 0.004).

### Subgroup analysis

**Fig. 4:**
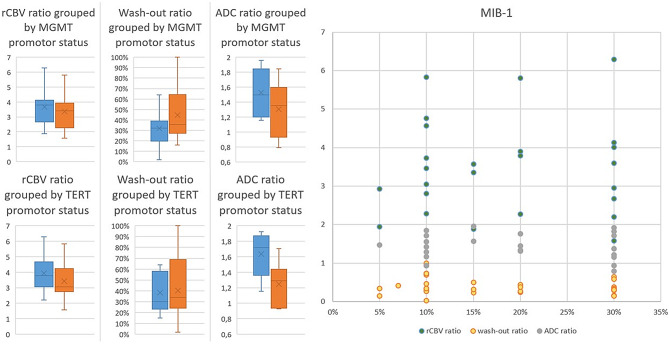
Subgroup analysis of patients with glioblastoma for O6-methylguanine-DNA methyltransferase (MGMT) status (blue-no methylation; orange-methylation), telomerase reverse transcriptase (TERT) mutation (blue-no mutation; orange-C228T) and a distribution chart of the Mindbomb E3 Ubiquitin Protein Ligase 1 (MIB-1) proliferation status in per cent. Box: interquartile range (IQR), which spans from the lower quartile (Q1) to the upper quartile (Q3). Whiskers: Indicate the minimum and maximum data range, excluding outliers. Median line: median. X: arithmetic mean

Figure 4 shows boxplots comparing the key analyzed properties of glioblastoma subgroups. The mean ADC ratios of patients with glioblastoma exhibiting the TERT promoter C228T mutation (mean 1.25 ± 0.25) and those with wild type (mean 1.63 ± 0.27) differed significantly. No significant differences were found regarding TERT promoter or MGMT promoter status for wash-out ratio or rCBV ratio.

**Fig. 5:**
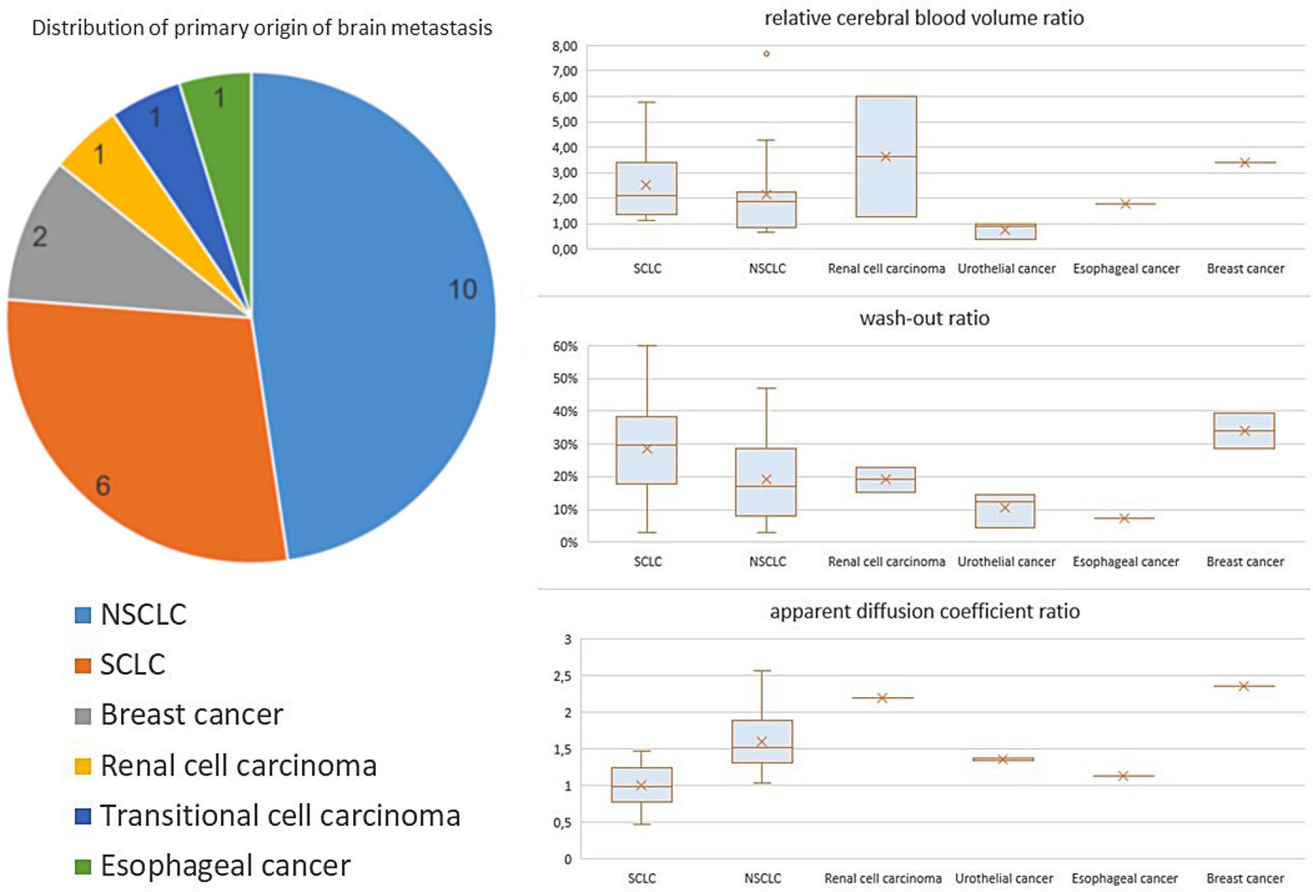
Subgroup analysis of patients with brain metastases. Pie chart of the distribution of brain metastasis (left); boxplots of the analyzed parameters grouped by primary origin (right). Box: interquartile range (IQR), which spans from the lower quartile (Q1) to the upper quartile (Q3). Whiskers: Indicate the minimum and maximum data range, excluding outliers. Median line: median. X: arithmetic mean

Figure 5 highlights differences in the analyzed properties of brain metastasis. In contrast to the rCBV and the wash-out ratios, the ADC ratios of non-small cell lung cancer (1.59 ± 0.42) and small cell lung cancer (0.99 ± 0.27) differed significantly.

### Interrater-reliability

The intraclass correlation coefficient for the measured wash-out volumes indicated excellent reliability, with an ICC(2,*k*) = 0.946 and a 95% confidence interval of [0.80, 0.98]. In contrast, the measurements of rCBV and ADC ratios exhibited significantly greater variability, with a good reliability (ICC(2,*k*) of 0.89) [95% CI = 0.78 to 0.94] and moderate reliability (ICC(2,*k*) of 0.72) [95% CI = 0.37 to 0.88], respectively.

## Discussion

In our study of 43 patients, we identified significant differences between glioblastomas and brain metastases using wash-out maps, enhancing the diagnostic accuracy in distinguishing these entities. This study represents the first systematic application of this method during the initial diagnostic work-up. A statistically significant difference in the wash-out ratio (*p* < 0.001) was observed, most likely due to a generally faster uptake and clearance of gadolinium in glioblastoma cells compared to metastatic tumor cells. Receiver operating characteristic (ROC) analysis revealed an optimal threshold for the wash-out ratio of 26%, yielding a sensitivity of 64% and a specificity of 79% for differentiating between the two tumor types. The combined parameter (rCBV ratio × wash-out ratio) performed even better, achieving a sensitivity of 76% and a specificity of 80% (AUC = 0.79) at an optimal threshold of 0.58. Additionally, the ICC was excellent for the wash-out maps, whereas greater variability was observed for rCBV.

These findings further suggest that the timing of the T1-weighted sequence following contrast administration is crucial for optimal visualization of mass lesions and may need to be adapted depending on the tumor type. Therefore, incorporating such a protocol at the time of the initial diagnosis should be considered, not only to improve comparability with subsequent preoperative imaging but also to ensure an accurate assessment of the tumor’s full extent.

Our results reveal that wash-out maps do not reflect the same information as MR perfusion and therefore have their own distinct diagnostic value.

Accordingly, the combined use of wash-out maps and rCBV may enhance diagnostic confidence and accuracy in differentiating glioblastoma from brain metastases at the time of initial presentation.

Similar approaches already exist that generate maps using two T1-weighted sequences to distinguish tumor recurrence from radiation necrosis [19]. Another method, TRAMs (Treatment Response Assessment Maps) [15, 20, 21], employs T1 weighted sequences with substantially greater temporal spacing. A study has already shown that the ideal time point for the first contrast-enhanced T1-weighted sequence using TRAMs is 15 min after contrast injection for metastases, whereas for glioblastomas, it is only 5 min [22].

It has already been demonstrated in a study that this late contrast agent behavior may differ from MR perfusion [10]. These findings suggest that this method offers a new diagnostic tool for differentiating tumor entities, which could be especially useful in clinically challenging scenarios, such as cases involving multifocal lesions [6, 23] and solitary brain metastases [24].

Despite our small sample size, we observed significant differences in the ADC ratios concerning the TERT promoter mutation. However, this association is not firmly established in the literature. While one publication successfully predicted the mutation status using a residual convolutional neural network and DWI [25], another study found no differences in the measured ADC values [26].

A recent study reported a CBV AUC of up to 0.85 for distinguishing between glioblastomas and solid brain metastases [27]. The lower AUC values observed for MR perfusion (0.80) and wash-out maps (0.73) may be attributed to the clinical, retrospective setup of the study and the inclusion of small lesions (3 × 3 × 3 mm) in our measurements. Smaller lesions are more susceptible to partial volume effects, which can compromise the accuracy of (perfusion and diffusion) measurements [28]. We also noted a different cut-off value for rCBV (2.23) compared to other previously published studies. For example, a study from Turkey found an optimal cut-off of 3.9 [24]. This is primarily dependent on the MRI protocol and scanner used; however, it may also be due to the 2021 revision of the glioblastoma definition [29].

One potential pitfall of the proposed technique is the presence of micrometastases. Although these may show focal areas of wash out, the lesions are often too small to be reliably measured. Another important consideration is the need for strict adherence to the imaging protocol, particularly regarding the timing between contrast administration and the acquisition of T1 weighted sequences. Deviations, such as delays caused by patient-related factors like panic attacks or movement, can result in time shifts that affect the appearance and interpretation of the wash out maps. Minor deviations may cause subtle differences, but a prolonged delay before the first sequence can lead to substantial distortion of the curve. Furthermore, highly vascularized metastases may exhibit contrast kinetics similar to glioblastomas, which likely accounts for some of the outliers observed in our statistical analysis. These considerations highlight the importance of consistent imaging protocols and cautious interpretation within the clinical context.

Our study suggests that, when evaluating an intra-axial brain tumor suspicious for glioblastoma or brain metastasis, diagnostic confidence may be improved by first asking: Is the rCBV ratio less than 2.2? Then, by interpreting the washout maps: Is the washout ratio less than 0.26, and is the product of the washout ratio and rCBV less than 0.58? This stepwise approach may help refine the differential diagnosis at the time of initial imaging.

### Outlook

Incorporating new characteristics (e.g., wash-out) into radiomic analysis could potentially improve diagnostic accuracy in brain tumor assessments [30, 31]. When combined with AI algorithms, particularly machine learning models, these features can be analyzed to predict tumor characteristics, genetic profiles, and patient outcomes [32].

### Limitations

One limitation is the retrospective nature of the study. The limited sample size, along with the inclusion of small lesions (3 × 3 × 3 mm), imposes constraints on the statistical power of our findings. This threshold was set to exclude micrometastases, as they are often only visible in the late-enhanced wash-out images and typically exhibit a wash-out ratio close to zero. Including them would artificially lower the average wash-out ratio value for metastases.

A further limitation is the heterogeneity of the metastasis group, as the proliferation index does not play the same role in all entities. Furthermore, prior systemic treatment may also have an influence, as we used only the initial diagnosis of brain metastases as the inclusion criterion. An already administered systemic therapy against the primary tumor was no exclusion criteria. Despite their frequency, cerebral melanoma metastases were not included in the study.

The average volume difference between GBM and metastases is significant and could influence the measured values, particularly MR perfusion, which is susceptible to partial volume effects. To prevent this, we established the minimum lesion size and used the wash-out ratios.

## Conclusion

The combination of late-enhancement and wash-out can serve as an additional MRI marker in brain tumor diagnostics. Especially, the combination of rCBV and wash-out may enhance the precision of differential diagnosis at the time of initial presentation. However, similar to MR perfusion, there remains an overlap between glioblastomas and metastases, which prevents definitive differentiation.

In an era where radiomics and AI are gaining increasing importance, the integration of additional markers holds significant potential to improve diagnostic accuracy.

**Table 1:** Mean ± standard deviation of the measurements

Subgroup	Glioblastoma (29 lesions)	Brain metastasis (53 lesions)	*p* value
Contrast enhancing tumor volume	12,992 ± 18,089 mm³	4,488 ± 6,756 mm³	< 0.001
rCBV ratio	349 ± 125%	232 ± 181%	0.05
Wash-out volume (mm³)	3,836 ± 5,808 mm³	929 ± 1,608 mm³	0.01
Wash-In volume (mm³)	9,156 ± 13,005 mm³	4,489 ± 6,756 mm³	0.08
Wash-out ratio	39 ± 21%	23 ± 15%	0.03
rCBV ratio × wash-out ratio	128 ± 81%	47 ± 37%	0.0001
ADC ratio	141 ± 33%	138 ± 47%	0.09

mm³ – cubic millimeter; rCBV –relative cerebral flood volume; ADC – apparent diffusion coefficient

## Data Availability

The datasets used and/or analyzed during the current study available from the corresponding author on reasonable request.

## Abbreviations

ADC: Apparent Diffusion Coefficient
AI: Artificial Intelligence
ASL: Arterial Spin Labeling (MR perfusion)
AUC: Area under Curve
CI: Confidence interval
DSC: Dynamic Susceptibility Contrast (MR perfusion)
DWI: Diffusion-Weighted Imaging
EPI-DWI: Echo-planar diffusion-weighted imaging
FLAIR: Fluid-attenuated inversion recovery
FLIRT: FMRIB’s Linear Image Registration Tool
ICC: Intraclass correlation coefficient
IDH: Isocitrate dehydrogenase
IQR: Interquartile range
MGMT: O6-methylguanine-DNA methyltransferase
MRI: Magnetic Resonance Imaging
MRS: Magnetic Resonance Spectroscopy
PACS: Picture Archiving and Communication System
PWI: Perfusion-weighted imaging
rCBV: Relative Cerebral Blood Volume
ROC: Receiver Operating Characteristic
SD: Standard Deviation
TERT: Telomerase reverse transcriptase
TRAMs: Treatment Response Assessment Maps
